# Insights into the Dual Anticancer and Antibacterial Activities of Composites Based on Silver Camphorimine Complexes

**DOI:** 10.3390/jfb15090240

**Published:** 2024-08-23

**Authors:** Joana P. Costa, Sílvia A. Sousa, Jorge H. Leitão, Fernanda Marques, Marta M. Alves, M. Fernanda N. N. Carvalho

**Affiliations:** 1 Centro de Química Estrutural, Institute of Molecular Sciences and Departamento de Engenharia Química, Instituto Superior Técnico, Universidade de Lisboa, Avenida António José de Almeida, n.º 12, 1000-043 Lisboa, Portugal; joanavcosta@tecnico.ulisboa.pt; 2 iBB-Institute for Bioengineering and Biosciences, Department of Bioengineering, Instituto Superior Técnico, Universidade de Lisboa, Avenida António José de Almeida, n.º 12, 1000-043 Lisboa, Portugal; sousasilvia@tecnico.ulisboa.pt (S.A.S.); jorgeleitao@tecnico.ulisboa.pt (J.H.L.); 3 C2TN-Centro de Ciências e Tecnologias Nucleares and Departamento de Engenharia e Ciências Nucleares, Instituto Superior Técnico, Universidade de Lisboa, EN 10, km 139.7, Bobadela, 2695-066 Loures, Portugal; fmarujo@ctn.tecnico.ulisboa.pt

**Keywords:** silver camphorimine complexes, HAp composites, anticancer and antibacterial activities, HOS cell line, osteosarcoma

## Abstract

Hydroxyapatite (HAp) is a widely used biocompatible material in orthopedic composite preparations. However, HAp composites that exhibit both anticancer and antibacterial activities through bioactive coordination complexes are relatively rare. To explore orthopedic applications, we blended several silver camphorimine compounds with HAp to create [Ag(I)] composites. All compounds [Ag(NO_3_)(L)_n_] (n = 1,2) based on camphorimine (L^A^), camphor sulfonimine (L^B^) or imine bi-camphor (L^C^) ligands demonstrated significant cytotoxic activity (IC_50_ = 0.30–2.6 μg_Ag_/mL) against osteosarcoma cancer cells (HOS). Based on their structural and electronic characteristics, four complexes (**1**–**4**) were selected for antibacterial evaluation against *Escherichia coli*, *Burkholderia contaminans*, *Pseudomonas aeruginosa*, and *Staphylococcus aureus*. All complexes (**1**–**4**) revealed combined anticancer and antibacterial activities; therefore, they were used to prepare [Ag(I)]:HAp composites of 50:50% and 20:80% weight compositions and the activities of the composites were assessed. Results showed that they retain the dual anticancer and antibacterial characteristics of their precursor complexes. To replicate the clinical context of bone-filling applications, hand-pressed surfaces (pellets) were prepared. It is worth highlighting that no agglutination agent was necessary for the pellet’s consistency. The biological properties of the so-prepared pellets were assessed, and the HOS cells and bacteria spreading on the pellet’s surface were analyzed by SEM. Notably, composite **4B**, derived from the bicamphor (L^C^) complex [Ag(NO_3_)(OC_10_H_14_N(C_6_H_4_)_2_NC_10_H_14_O)], exhibited significant anticancer activity against HOS cells and antibacterial activity against *P. aeruginosa*, fostering potential clinical applications on post-surgical OS treatment.

## 1. Introduction

Osteosarcoma (OS) is a primary malignant bone tumor that mainly affects children and young adults [1,2]. Surgery, followed by chemotherapy to control micro-metastasis after surgical resection, is the strategy typically used for the management of OS [3]. Advances in these treatments, such as targeted therapies that signalize and destroy cancer cells or neoadjuvant chemotherapy, that control tumor growth before surgery have transformed this fatal disease into a less life-threatening condition, resulting in an increased survival rate for some patients. Nevertheless, the high tendency of OS to metastasize, especially in the lungs, and the development of chemotherapy resistance with associated severe side effects are still major concerns [4]. Consequently, the control of cancer metastasis and infections in patients becomes a challenge to decrease morbidity and mortality in patients with OS. The increasing complexity of the disease requires a multidisciplinary approach, including exhaustive chemotherapeutic treatments and control of microbial infections due to immunity impairments. In severe OS, surgery and bone reconstruction are often associated with potential benefits for patients, like restoring function, tumor removal or pain, reduction the risk of metastasis and, overall, increasing the chances of survival [5,6].

Hydroxyapatite (HAp) is often used for bone reconstruction due to its biocompatibility and adhesion properties [7,8,9,10]. However, its brittleness and low fracture toughness limit load-bearing applications, especially in patients that require extensive bone resection. Additionally, HA’s inability to prevent cancer recurrence further limits its effectiveness in treating OS, where both mechanical durability and anticancer functionality are crucial for successful outcomes.

This is of special concern for oncologic patients, who are highly vulnerable to infections caused by opportunistic bacteria or fungi due to their immune-depressed systems. In fact, nosocomial bacterial infections are the major complication of orthopedic implant surgery, resulting in high morbidity and extended hospital stays [11]. Biofilm formation on the implants is the major cause of the resistance of these bacteria to the currently used antibiotics and compromises the host immune response. What constitutes bone implant-associated infections, those caused by Gram-negative *Pseudomonas aeruginosa*, or Gram-positive *Staphylococcus aureus* are the most frequent and the most difficult to treat due to multidrug resistance [12,13,14,15]. *Burkholderia contaminans* and *Escherichia coli* are bacterial strains that represent a considerable threat due to lung and other organ infections associated with bone cancer metastasis [16].

To increase the success of OS treatments, a multidisciplinary approach able to face the above-mentioned risks is necessary. A potentially beneficial strategy is combining anticancer and antimicrobial agents [17] with the well-known biocompatibility of HAp, since the so-generated biologically active composites may reinforce the oncologic treatment and concomitantly reduce the occurrence of metastasis and infections. To bind composites, agglutinating agents (e.g., polymers) are commonly required [18,19]. Some attempts to functionalize biocompatible materials with commercial antibiotics aimed at preventing *post*-surgical infections associated with implants for bone reconstruction have been made [18,20,21,22,23]. However, the increasing resistance of the abovementioned microorganisms to commercially available antibiotics requires new agents (molecules or nanoparticles) acting by different mechanisms to defeat microorganism proliferation and/or biofilm formation [17,24,25,26]. Under such a purpose, coordination compounds are feasible options due to the presence of the metal that may enable specific types (e.g., redox) of interactions with microorganisms.

A strategy often reported has been the functionalization of hydroxyapatite (Ca_10_(PO_4_)_6_(OH)_2_) with metals (Ag, Ni, Si, Se, Zn, others) having complementary bioactive properties [27,28,29,30,31,32,33,34]. However, scarce examples exist [35] of bioactive coordination compounds being used to prepare composites of HAp, or other bone reconstruction agents while acting as anticancer/antimicrobial agents [20,21,28].

Within our previous research work, the combined anticancer and antimicrobial activities of several Ag(I) camphorimine complexes have been reported against ovarian cancer cells and a set of microbial strains [36,37,38]. Under the scope of orthopedic applications, HAp composites were now prepared based on the Ag(I) camphorimine complexes. The coordination polymer character of the complexes enables agglutination with HAp without the need for extra agglutinating agents. The biological properties of the new composites were evaluated against the human HOS osteosarcoma cell line and several bacterial strains (*E. coli ATCC25922*, *B. contaminans* IST408, *P. aeruginosa* 477, *S. aureus Newman*).

The herein results pertaining to the characteristics of HAp composites functionalized with Ag(I) camphorimine complexes showed that they combine anticancer (HOS cells) and antibacterial properties while enhancing the HAp agglomeration character.

## 2. Results and Discussion

The results obtained by us under the study of the anticancer and/or antimicrobial properties of coordination compounds based on camphorimine ligands (L) [39] showed that the activities of the complexes can be modulated through the selection of the metal center (e.g., Ag(I), Au(I), Cu(I), Cu(II)) and the camphorimine substituent (Y, R, or Z, Table 1).

In this study, the anticancer activity of a selection of silver camphorimine complexes ([Ag(I)]) was assessed against HOS osteosarcoma cells. In parallel, the antibacterial properties of complexes **1** and **2** against *E. coli* ATCC25922, *B. contaminans* IST408, *P. aeruginosa* 477 and *S. aureus Newman* strains were also assessed. This study also includes the results [40] previously obtained on the antibacterial activities of complexes **3**–**4** against the same bacterial strains.

Foreseeing orthopedic applications the preparation of bioactive [Ag(I)]:HAp composites were initiated aiming at combining cytotoxic activity towards HOS osteosarcoma cells, and antibacterial properties against Gram-negative (*E. coli* ATCC25922, *B. contaminans* IST408, *P. aeruginosa* 477) and Gram-positive (*S. aureus* Newman) strains.

### 2.1. Cytotoxic Activity of the Ag (I) Camphorimine Complexes towards HOS Osteosarcoma Cells

Three sets of Ag(I) camphorimine compounds—[Ag(I)]: [Ag(NO_3_)(OC_10_H_14_NY)] (Y = NH_2_, **1**), [Ag(NO_3_)(OC_10_H_14_NY)_2_] (Y = 3-OHC_6_H_4_, **2**; Y = OH; Y = C_6_H_5_), [Ag(NO_3_){(OC_10_H_14_N)_2_Z}] (Z = 4-(C_6_H_4_), **3**; Z = 4-(C_6_H_4_)_2_, **4**) and [Ag(NO_3_)(O_2_SNC_10_H_14_NR)_2_] (R = C_6_H_5_; R = C_6_H_4_NH_2_)) (Table 1) based on different types of camphor-derived ligands (L^A^, L^B^ or L^C^) were synthesized using previously reported procedures [41]. The bulk purity of all complexes was confirmed by elemental analysis and ^1^H and ^13^C NMR, and their activity against HOS cancer cells was evaluated by the MTT assay based on the metabolic activity of viable cells (see Section 6).

**Figure 1:**
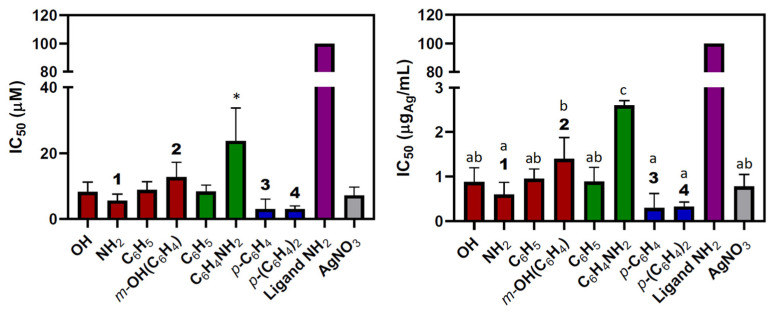
IC_50_ values for [Ag(NO_3_)L_n_] complexes (L = L^A^, red; L^B^, green; L^C^, blue), ligand OC_10_H_14_NNH_2_ (purple) and silver nitrate (gray), shown versus molecular concentration (left, µM) and silver weight (right, µg_Ag_/mL). Bars represent average errors, and a one-way ANOVA was used to detect differences, followed by multiple comparisons test, (*) denotes statistically significant differences in the left-hand plot, while letters indicate significant differences in the right-hand plot for *p*-value < 0.05.

The IC_50_ values (Figure 1) show that all complexes efficiently inhibit HOS cellular viability, no matter the type of camphorimine ligand (L^A^, OC_10_H_14_NY; L^B^, O_2_SNC_10_H_14_NR; L^C^, OC_10_H_14_N)_2_Z) or the metal-to-ligand ratio (Table 1). In contrast, the free camphorimine ligands display very low cytotoxic activity (e.g., OC_10_H_14_NNH_2_: IC_50_ > 100 µM) under the same experimental conditions.

The lowest IC_50_ values (highest activity) were obtained for the bi-camphor complexes [Ag(NO_3_)(L^C^)] (**3**, **4**) (Figure 1) while the highest IC_50_ value (23.7 ± 10 µM, i.e., 2.6 ± 0.1 μg_Ag_/mL) was obtained for the camphor sulfonimine complex [Ag(NO_3_)(L^B^)_2_] (L^B^, R = PhNH_2_).

The IC_50_ values for the complexes with different types of ligands [Ag(NO_3_)(L)_2_] (L = L^A^, Y = C_6_H_5_, 8.8 ± 2.5 µM or L^B^; R = C_6_H_5_, 8.3 ± 2.0 µM) and equal imine substituents (Y = R) do not differ appreciably. In contrast, the IC_50_ values for the complexes with the same type of ligand (L^B^) but different imine substituents (e.g., R = C_6_H_5_: 8.3 ± 2.0 µM or R = C_6_H_4_NH_2_: 23.7 ± 10.0 µM) differ considerably, showing that the imine substituent has a higher impact on the cytotoxicity of the complexes than the bicyclic (L^A^) or tricyclic (L^B^) character of the camphor-derived ligand, as previously observed for the A2780 and OVCAR3 ovarian cancer cells [36].

The IC_50_ values versus the silver content (µg_Ag_/mL) are ca. one order of magnitude lower than those (µM) of the complexes (Figure 1).

The IC_50_ value of the metal precursor (AgNO_3_) does not differ much from those of the complexes, in agreement with the cytotoxic activity relying on the metal ion, as previously reported [37]. Nevertheless, complexes **1**, **3**, and **4** display lower IC_50_ values (Figure 1) than silver nitrate. The low IC_50_ values (Table 1) displayed by complexes **3** and **4** are attributed to the characteristics of the bi-camphor ligands (L^C^) that can better enable the cytotoxic activity of the complexes. These IC_50_ values measured (Table 1) for complexes **3** (3.1 ± 3.0 μM) and **4** (3.1 ± 0.9 μM) are lower than those of actinomycin D (9 μM) [42] or denosumab (34.8 μM) [43], which are drugs presently under use to treat OS.

### 2.2. Cytotoxic and Antibacterial Activities of [Ag(I)]:HAp Composites

The cytotoxic activity of the Ag(I) camphorimine complexes against HOS cells (Table 2) encouraged a deeper insight into their behavior, foreseeing possible orthopedic applications. Thus, a selection of complexes ([Ag(NO_3_)(L^A^)] (Y = NH_2_, **1**), [Ag(NO_3_)(L^A^)_2_] (Y = *m*-OH(C_6_H_4_), **2**), [Ag(NO_3_)(L^C^)] (Z = *p-*(C_6_H_4_), **3**), [Ag(NO_3_)(L^C^)] (Z = *p-*(C_6_H_4_)_2_, **4**)) was used for preparation of [Ag(I)]:HAP composites with compositions 50:50% and 20:80% weight ratios (see Section 6).

**Figure 2:**
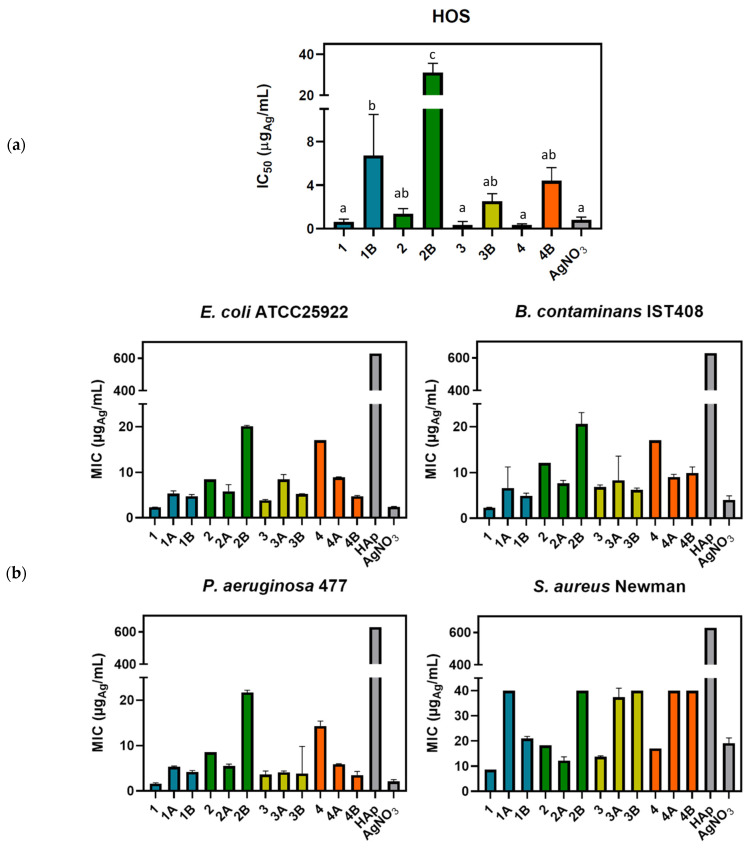
Graphic representation of data for complexes (**1**–**4**) and composites ([Ag(I)]:HAp, 50:50%, **1B**–**4B**): (**a**) anticancer activities (IC_50_); (**b**) antibacterial activities (MIC). All values are referred to in μg_Ag_/mL to facilitate comparison. Bars represent average errors, and a one-way ANOVA was used to detect differences, followed by multiple comparisons test, where letters indicate significant differences for *p*-value < 0.05.

The high percentage of HAp in the 20:80% [Ag(I)]:HAp composites interfered with the MTT colorimetric method, preventing the accurate determination of their cytotoxic activity. Consequently, solely the activity of the [Ag(I)]:HAp composites with 50:50% weight ratios (Table 2) against the HOS cancer cells is reported. To facilitate perception, a graphical representation of IC_50_ and MIC values is displayed (Figure 2).

The herein results show that the cytotoxic activities of the composites, as expected, are typically one order of magnitude lower than those of the precursor complexes (Table 2) and follow the trend in their precursor complexes.

To obtain insights into the antibacterial activity of composites (**1A**–**4A** and **1B**–**4B**) towards the bacterial strains *E. coli* ATCC25922, *B. contaminans* IST408, *P. aeruginosa* 477, and *S. aureus* Newman, their minimum inhibitory concentrations (MIC) were evaluated. The results (Table 2, Figure 2b) show that the composites have lower antibacterial activity (higher MIC values) than the precursor complexes, except for composite **2A,** which displayed higher activity than complex **2** for the four bacterial strains under study (Figure 2b). Such a trend suggests that the substituent (*m*-OH(C_6_H_4_)) at the camphorimine ligand (L) prompts a structural organization into the [Ag(I)]:HAp composite (20:80%) that enhances the inhibition of bacterial activity. Such behavior is attributed to strong interactions being established between complex **2** and Hap, thus reducing the complex-to-complex interactions. This hypothesis is somehow corroborated by the fact that the effect is absent in the [Ag(I)]:HAp composite (**2B**, 50:50%) with a considerably lower quantity of HAp. From the composites under study, **2B** displays the less encouraging results. Composites **1B**, **3B,** and **4B** efficiently combine anticancer and antibacterial activities. Overall, the complexes and composites behave better against Gram-negative than Gram-positive (*S. aureus* Newman) bacteria.

## 3. Physiochemical Characterization of Ag(I) Complexes and [Ag(I)]:HAp Composites

**Figure 3:**
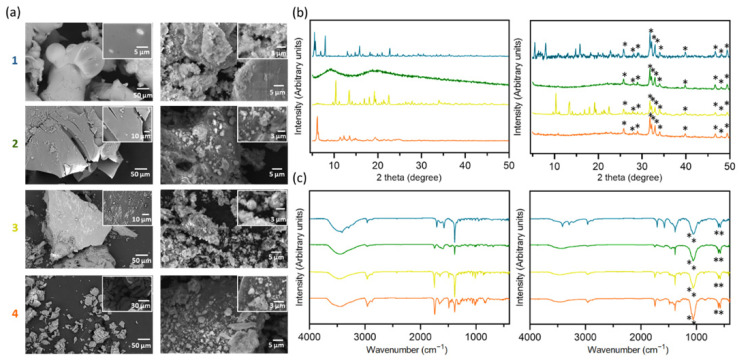
Physicochemical characterization of the complexes (**1**–**4**, left) and composites (**1B**–**4B**, right. **1**, **1B**—blue; **2**, **2B**—green, **3**, **3B**—yellow and **4**, **4B**—orange. (**a**) SEM images of complexes (left) and respective composites (right) with zoom on the up-right corner; (**b**) PXRD diffractograms from complexes (left) and respective composites (right) with HAp signals marked (*); (**c**) FTIR spectra from complexes (left) and respective composites (right) with HAp bands marked (**).

The selected complexes (**1**, **2**, **3**, **4**) and their derived composites [Ag(I)]:HAp, 50:50% weight composition) were physiochemically characterized by Scanning Electron Microscopy (SEM), Powder X-Ray diffraction (PXRD), Fourier Transform Infrared (FTIR) (Figure 3) and Energy Dispersive Spectroscopy (EDS).

As shown in Figure 3a, the particles that form the complexes are homogeneous. In **1** and **3**, the bright spots observed are round-shaped in complex **1**, while in complex **3**, they are needle-like-shaped. Carbon, nitrogen, hydrogen, silver, and oxygen were identified in the samples by Energy Dispersive Spectroscopy (EDS) analysis, as previously described for related camphorimine complexes [45]. A detailed analysis of the dark and bright spots observed in the powders of complexes **1** and **3** shows no difference in silver content.

EDS analysis of the composites (**1B**, **2B**, **3B**, **4B**) confirmed the additional presence of calcium and phosphorus from HAp. Composites **1B** and **3B** are homogeneous, with Ag(I) complexes and HAp components evenly distributed. In contrast, composites **2B** and **4B** display a compact structure covered by blocks of the complex and HAp.

Visually, only complex **1** changed color in the composite; it went from gray to black; all the others kept the original color.

The crystallinity of the complexes and composites was analyzed by PXRD (Figure 3b). The comparison of the PXRD diffractograms of complexes and their derived composites shows that the crystallinity of complexes (**1**, **3**) was preserved in composites (**1B**, **3B**), and the amorphous nature of complex **2** was kept in composite (**2B**). A different pattern was observed for complex **4**, which lost crystallinity and became amorphous within composite **4B**. Such a loss of crystallinity is attributed to the characteristics of the imine bi-camphor ligand with a biphenyl spacer binding the two chiral camphor moieties. The versatility of the biphenyl spacer allows the molecule to rotate and rearrange as a disordered system during the preparation of the composite (ethanol: dissolution/evaporation along with mechanical stirring).

FTIR analysis was undertaken to ascertain whether the complexes’ integrity is maintained in the composites. The data obtained for composites (**1B**, **2B**, **3B**, Figure 3c) are consistent with the integrity of the complexes being retained in the composites since the functional bands (ν_C=O_ and ν_NO3_) are observed at identical wavenumber values, although with less intensity. In contrast, in the FTIR spectrum of composite **4B**, the ν_CO_ and ν_NO3_- bands switched intensities. Such behavior is attributed to structural changes in the complex inside the composite, in agreement with the loss of crystallinity identified by PXRD. The FTIR bands characteristic of HAp (1086, 1061, 604, and 572 cm^−1^) were observed in all the composites.

## 4. Biological Activity of Pellets Obtained from [Ag(I)]:HAp Composite

To mimic the conditions used by surgeons during bone filling, pellets from composites were gently shaped to simulate clinical environments to obtain insights into the biological response of the [Ag(I)]:HAp composites to manual pressure. Such an approach is innovative since reports on the study of biofilm formation on pellets from HAp composites are scarce and have been typically reported to involve powders of doped HAp composites or coatings [46,47,48].

No pellets from 20:80% [Ag(I)]:HAp composites could be obtained due to the lack of aggregation and poor tenacity of the material. In contrast, the pellets obtained from [Ag(I)]:HAp (50:50%) composites revealed excellent aggregation. No agglutinating agent was necessary due to the physical interaction between the complexes and HAp that exempts the need for external aggregators. Such behavior is considered an added value concerning orthopedic applications.

The anticancer and antibacterial properties of the [Ag(I)]:HAp (50:50%) composites and HAp pellets were assessed. For a better comprehension of the process, the analysis of the pellet surface was made upon exposure to cancer cells or bacteria.

### 4.1. Pellets’ Superficial Behavior

**Figure 4:**
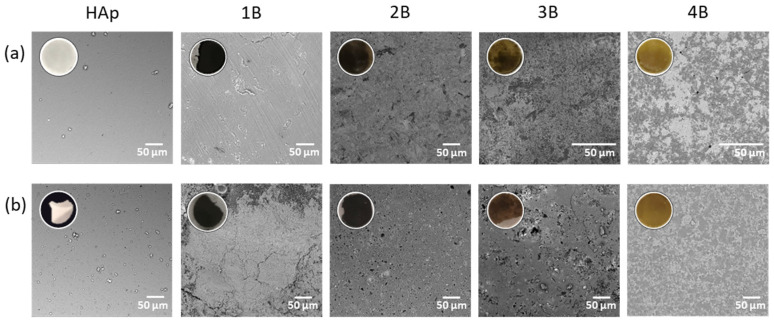
Pellets morphology. SEM images of the pellet surface of HAp and composites (**1B**–**4B**) (**a**) before and (**b**) after immersion in cell culture medium DMEM and fixation. Insets are optical images of pellets before (first row) and after (second row) 24 h incubation in culture medium.

Initial inspection of the white HAp pellet showed it was very frail, in contrast with pellets **2B**, **3B,** and **4B** that were compact and firm. Pellet **1B** was difficult to detach from the press; its texture was comparable to that of hard plastic, dark with a brilliant surface. Pellet **2B** presented a dark brown color, **3B** a dark yellow, and **4B** a yellow color (Figure 4a). Consequently, the pellets maintained the same color as the composite powder.

To screen the behavior of the pellets in contact with body fluids, they were immersed in Dulbecco’s Modified Eagle’s Medium (DMEM), the medium used for supporting the growth of HOS cells. Upon 24 h of immersion, the visual inspection revealed the integrity of all pellets obtained from the composites (**1B**–**4B**). On the contrary, the pellets prepared solely from HAp were desegregated. Since cell fixation steps were necessary for the subsequent study, which involved cancer cells and bacteria spreading over the pellet surface, an investigation was conducted to assess the integrity of the pellets after the fixation process. SEM images were obtained and analyzed (Figure 4). Images revealed that the surface of the pellets remained homogeneous after the immersion and fixation steps (Figure 4). The HAp pellet showed loose pieces (Figure 4b), but the surface stayed uniform. Pellet **1B** presented slits, **2B** and **3B** displayed a few more holes after immersion, while at pellet **4B**, no visible alterations could be detected. The color of the pellets showed negligible differences between the two stages.

The pellets’ consistency supports the ability of the complexes (**1**–**4**) to act as agglomerating agents, given the lack of an external binder.

### 4.2. Cell Cytotoxic Response

**Figure 5:**
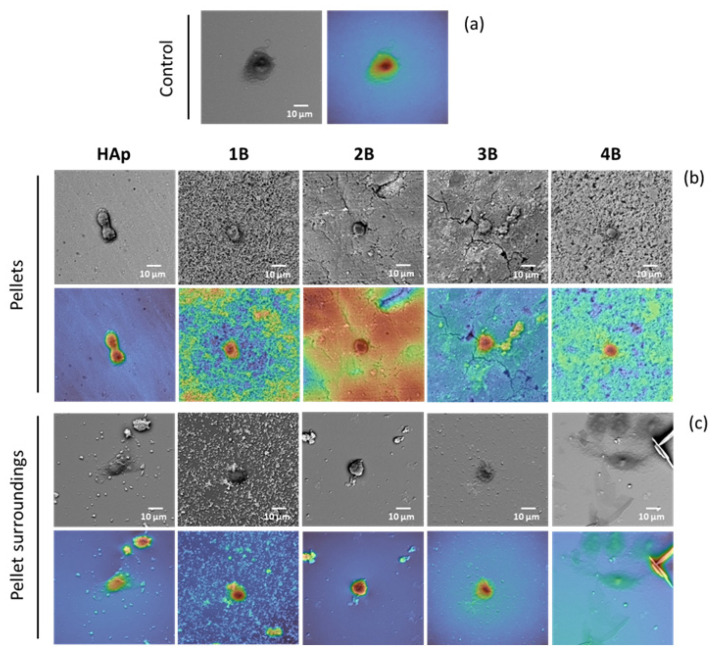
HOS cells morphology settled on th4e pellets surface and its surroundings. SEM images: (**a**) control HOS cells settled; (**b**) HAp and **1B**–**4B** composites pellets surface; (**c**) lamella surrounding the pellets. Images were taken after incubation (24 h) of the cells in culture medium, in the presence of the pellets.

To investigate the effects of the composite surface on the morphology of the HOS osteosarcoma cancer cells spreading, hand-made pellets of composites **1B**–**4B** were incubated with the cells (HOS) for 24 h. SEM (Figure 5) analysis of the surface showed cells with typical spheroid shapes on either the pellet surface (Figure 5b) or the surface surrounding the pellet (Figure 5c) [49]. For comparison purposes, control images were collected from cells spread over glass (Figure 5a).

The analysis of the surface of the pellets and lamella (upon 24 h exposure) shows HOS cells adherent to the surface of the pellet as well as cells in the lamella surrounding the pellet. However, the morphologies are different from one another (Figure 5).

The morphology of the cells adhered to composites **2B**, **3B,** and HAp pellets is similar, suggesting that complexes allowed cells to actively phagocytize HAp (Figure 5b). A different behavior was evidenced by pellets **1B** and **4B** that show no explicit HAp uptake by cells, suggesting that somehow complexes **1** and **4** impaired it.

On the surrounds of HAp, **1B** and **2B** pellets (Figure 5c) dispersed HAp fragments along with spheroid cells, suggesting that the cells were up taking these loose particles [49]. The release of HAp particles into the pellet’s surroundings followed the order **1B** > HAp > **2B**. These three types of pellets were brittle 24 h after immersion, whereas those derived from complexes **3** and **4** remained unbroken (Figure 4 and Figure 5b). The disaggregation pattern observed for composites **1B** and **2B** can be attributed to the hydrogen bonding characteristics of the camphorimine substituents (Y, Z, Table 1) at the complexes (i.e., NNH_2_ in **1** and *m*-OHC_6_H_4_ in **2**) that increase hydrophilicity, facilitate interactions with the medium or cells, and prompt disaggregation of the pellets. It is noteworthy that composite **1B** developed a net around cells (Figure 5b), which we hypothesize to result from cells preferring interactions with the **1B** composite to those with bare HAp (Figure 5b). Such behavior may be relevant to enhancing the efficacy of this composite as an anticancer agent. From the systems under study, only composites **2B** and **3B** favored mineralization by osteosarcoma cells.

A flat cell morphology similar to the control was seen in the surroundings of the **3B** and **4B** pellets, where no loose HAp fragments could be depicted (Figure 5c).

Pellets obtained from composites **3B** and **4B** were chosen for further antibacterial assay and morphological analysis because the HOS cancer cells showed distinct morphology when incubated with those pellets, while their precursor composites behave similarly in terms of anticancer and antibacterial activities (Figure 5).

### 4.3. Antibacterial Response

**Figure 6:**
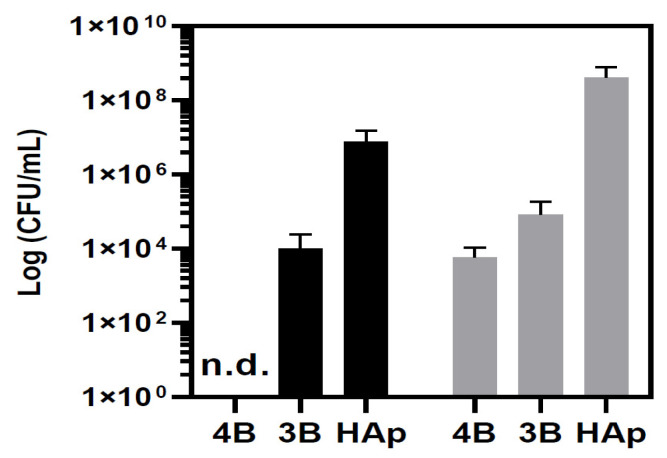
Antibacterial activity for the pellets of the composites **3B**, **4B**, and HAp towards *P. aeruginosa* 477 (black bars) and *S. aureus* Newman (gray bars). The number of colonies adherent to the pellets was estimated by serial dilution and spot inoculation (n.d.—non detected). The error bars represent the mean (±SD) of two independent experiments.

The antibacterial activity of the pellets obtained from [Ag(I)]:HAp composites (**3B** and **4B**) were evaluated by incubating the pellets with Mueller Hinton Broth liquid medium (MHB) inoculated with *P. aeruginosa* 477 or *S. aureus* Newman. As representative examples, one Gram-negative (*P. aeruginosa*) and one Gram-positive (*S. aureus*) strain were chosen. Pellets composed uniquely of HAp were used as controls. After incubation with *P. aeruginosa* and *S. aureus* on the surface of the HAp pellets, colonies of *S. aureus* (*ca*. 10^9^ CFUs/mL) and *P. aeruginosa* (*ca*. 10^7^ CFUs/mL) were detected. This trend was expected since HAp by itself was reported to have some anti-biofilm properties against *P. aeruginosa* [50]. Under the experimental conditions used for HAp pellets, those containing composites **3B** or **4B** demonstrated strong growth inhibition. No CFUs of *P. aeruginosa* were detected on the pellets of composite **4B**, in agreement with their excellent efficiency against colony formation. For the same bacterial strain, the pellets from composite **3B** showed a reduction of 3 log CFUs/mL (Figure 6).

In terms of activity against *S. aureus*, growth inhibition by the **3B** pellet was less pronounced than for *P. aeruginosa*. Yet, reductions of 4 and 6 logs in the *S. aureus* CFUs were detected in the pellets of composites **3B** or **4B**, respectively (Figure 6). Although pellets from both composites showed high activity against the bacterial strains under study, the **4B** pellet performed better. Since the HAp pellet (used as a control) showed no activity, the antibacterial activity of pellets containing composites **3B** and **4B** can be attributed to the Ag(I) camphorimine complexes (**3** and **4**).

**Figure 7:**
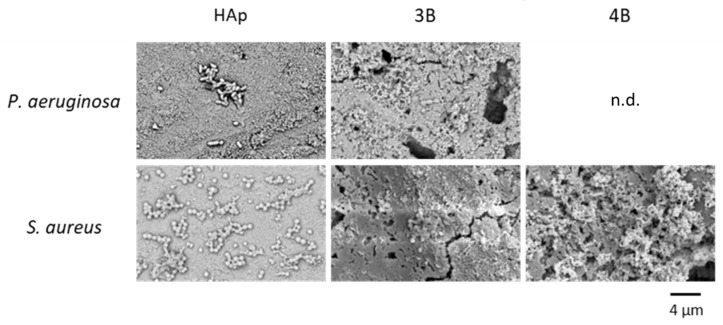
SEM images of the morphology of bacteria *Pseudomonas aeruginosa* 477 (first row) and *Staphylococcus aureus* Newman (second row) after incubation with HAp and [Ag(I)]:HAp composites **3B** and **4B** pellets (n.d.—non detected).

To gain insights into the bacterial morphology spreading over the pellets, SEM analysis was undertaken (Figure 7).

The SEM images of the surface of the HAp pellets showed cells of *P. aeruginosa* with their typical rod-like shape (bacilli) and *S. aureus* with a spherical-like shape (cocci), both forming biofilms [51,52,53,54]. In contrast, there was no sign of biofilm formation over pellets obtained from composites **3B** and **4B**, in agreement with the above-mentioned CFU results. The isolated cell morphology was similar to that observed over HAp pellet.

**Figure 8:**
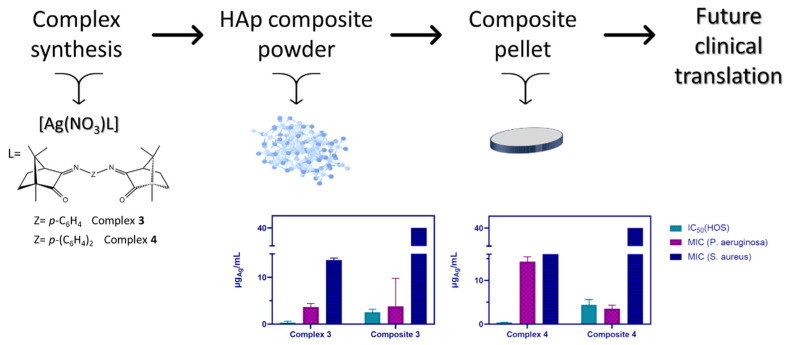
Schematic representation illustrating the various steps and results obtained for complexes **3**, **4** and their derived composites and pellets.

A schematic representation of the results obtained for complexes [Ag(NO_3_)(L^C^)] (**3**, **4**; L^C^: bi-camphor ligands) and their derived composites (**3B**, **4B**) and pellets is displayed in Figure 8. Data show that complexes and composites have significant potential either as anticancer or antibacterial agents. It is noteworthy that complexes **3** and **4** exhibit IC_50_ values (*ca*. 0.3 μg_Ag_/mL, Table 1) against HOS in the range of the OS anticancer agents in use [41,42] (e.g., Dactinomycin, IC_50_ = 0.42 μM) [55] and activity against common bacterial infections.

In terms of antibacterial efficacy against *P. aeruginosa*, complex **3** displays a MIC value (3.6 ± 0.8 μg_Ag_/mL, Table 2) ca. 4 times lower than that of complex **4**, evidencing its higher antibacterial activity. Against *S. aureus*, the two complexes display higher MIC values in the same range (Table 2), still consistent with high activity.

The composites (**3B**, **4B**), with half of the Ag(I) compared to the complexes, show a tenfold increase in the IC_50_ values against HOS (*ca*. 3–4 μg_Ag_/mL, Figure 8). In what concerns MIC values against *P. aeruginosa* assessed for the composites **3B** and **4B** distinct trends are observed. Composite **3B** and the precursor complex (**3**) display analogous MIC values (Table 2), while the MIC value for composite **4B** is considerably lower than that of **4** (Table 2). Conversely, the composite MIC values for *S. aureus* are both higher than those of the related complexes (Table 2).

The results obtained are consistent with a beneficial synergic interaction between the complexes (**3**, **4**) and HAp that enhances the activity of the composites against *P. aeruginosa*. Although composites inhibit cell proliferation, no such enhancement was detected for *S. aureus* (Figure 8).

## 5. Conclusions

The cytotoxic activities of a set of Ag(I) coordination compounds with three distinct types of camphor imine ligands were screened against osteosarcoma HOS cancer cells. Concomitantly, the antibacterial activity of the complexes against *E. coli* ATCC25922, *B. contaminans* IST408, *P. aeruginosa* 477, and *S. aureus* Newman bacterial strains was also assessed. The results showed that the complexes have excellent anticancer properties (HOS, IC_50_: 0.30–1.4 μg_Ag_/mL) combined with good antibacterial activities. From the eight complexes under study, four were selected (**1**–**4**) for further investigation based on their structural differences and combined anticancer and antibacterial activities.

Focused on potential orthopedic applications, composites ([Ag(I)]:HAp) with 20:80% and 50:50% weight compositions were successfully prepared. The evaluation of the biological properties of the 50:50% composites (**1B**–**4B**) demonstrates that they maintain the synergic anticancer/antibacterial activities of the precursor complexes. The loss of integrity of the 20:80% composites in the biological medium precluded accurate data, and their study was abandoned.

The IC_50_ values (HOS cells) obtained for composites **1B**–**4B** are higher (*ca*. one order of magnitude) than those assessed for their precursor complexes, but still significantly low from the point of view of orthopedic applications. In what concerns the MIC values, most of the composites display higher values than those of the complexes, although the differences depend on the complex and the bacterial strain. Significant exceptions to the tendency of loss of antibacterial activity by the composites, compared to the precursor complexes, are composites **3B** (3.8 ± 0.6 μgAg/mL) and **4B** (3.6 ± 0.8 μgAg/mL) that show MIC values against *P. aeruginosa* similar (**3B**) to the precursor complex (3, 3.6 ± 0.8 μgAg/mL) or even lower (**4B**) than complex **4** (14.3 ± 1.1 μgAg/mL). Such results are especially significant since *Pseudomonas aeruginosa* is a multi-resistant bacterium often responsible for infections in *post*-surgical orthopedic processes.

To support the potential use of the [Ag(I)]:HAp composites in bone filling processes, pellets were manually prepared, and their anticancer activity towards HOS cells, antimicrobial activity against *P. aeruginosa*, *S. aureus,* and morphological properties were assessed. The first outcome from the preparation and study of the pellets was their consistency, showing that the Ag(I) camphorimine complexes act as suitable agglomerating agents without the need for an external binder. In the biological medium, pellets maintained their integrity and homogeneity, as far as observed through morphological analysis. After 24 h incubation with HOS cancer cells, pellets showed different behaviors concerning morphology and cell interaction. When exposed to the two strains of bacteria, *P. aeruginosa* and *S. aureus,* the pellets preserved the antiproliferative efficiency of the complexes, as shown by CFU and SEM images.

The herein results consistently evidence that the dual anticancer and antibacterial properties of silver camphorimine complexes are retained in composites [Ag(I)]:HAp (50:50% in weight). Still more relevant is that active pellets that potentially mimic bone filling conditions can be obtained from the composites without the need for an additional agglutinating agent. The intrinsic nature of these complexes, structurally arranged as coordination polymers, qualifies them as effective aggregating agents for potential clinical applications.

From the composites under study, **4B** displays the highest HOS anticancer and antibacterial activity against *P. aeruginosa* which is one of the bacteria that causes the highest concern due to antibiotic resistance.

The overall reported results on the combined anticancer and antibacterial properties of composites and pellets derived from silver camphorimine complexes foster complementary studies to evaluate their use in clinical settings.

## 6. Experimental Section

### 6.1. Synthesis of Complexes, Composites, and Pellets

All the camphor imine complexes, except [Ag(NO_3_){SO_2_NC_10_H_13_N(C_6_H_4_NH_2_-4)}_2_]·^5^/_2_H_2_O were previously synthesized and characterized according to published procedures [56]. The synthesis, analytical, and spectroscopic data concerning complex [Ag(NO_3_){(SO_2_NC_10_H_13_N(C_6_H_4_NH_2_-4)}_2_]·^5^/_2_H_2_O are described below.

The complex and the composites were prepared under nitrogen using Schlenk and vacuum techniques. The FT-IR spectra were obtained from KBr pellets using a JASCO FT/IR 4100 spectrometer (JASCO Corporation, 2967-5 Ishikawamachi, Hachioji, Tokyo Japan). The NMR spectra (^1^H, ^13^C, DEPT, HSQC, and HMBC) were obtained from CD_3_CN and DMSO solutions using Bruker Avance II+ (300 or 400 MHz) spectrometers (Bruker BioSpin AG, Industriestrasse 26, Fällanden, Switzerland. The NMR chemical shifts are referred in TMS (δ = 0 ppm). The elemental analyses were obtained from the accredited IST-Analysis Laboratory (LAIST).

### 6.2. Complex

[Ag(NO_3_){SO_2_NC_10_H_13_N(C_6_H_4_NH_2_-4)}_2_]·^5^/_2_H_2_O—AgNO_3_ (85 mg; 0.50 mmol) and SO_2_NC_10_H_13_NC_6_H_4_NH_2_-4 (256 mg; 1.0 mmol) were stirred under vacuum for 30 min. Then, CH_3_CN (5 mL) was added, and the mixture was stirred for another 2 h. The suspension was filtered to remove reaction residues. Then, the solvent was partially evaporated, and the solution was placed in the fridge overnight. A dark red solid formed that was filtered off solution, affording the desired compound. Elem. Anal. for AgC_32_H_38_N_7_O_7_S_2_·^5^/_2_H_2_O Exp.: C, 45.5; N, 11.2; H, 4.9; S, 7.8. Calc.: C, 45.2; N, 11.5; H, 5.1; S, 7.5. IR (KBr, cm^−1^): 3485, 3384 (NH_2_); 1641 and 1623 (CN); 1384 (NO_3_); 1317 (SO_2_); 1161 (SO_2_). ^1^H NMR (300 MHz, CD_3_CN, δ ppm): 7.00 (d, *J* = 8.7 Hz, 2H); 6.71 (d, *J* = 8.7 Hz, 2H); 3.45, 3.23 (2d, *J* = 14 Hz, 2H); 3.17 (d, *J* = 4.4 Hz, 1H); 2.33–2.19 (m, 2H); 1.89–1.75 (m, 2H); 1.07 (s, 3H); 0.81 (s, 3H). ^13^C NMR (300 MHz, CD_3_CN, δ ppm): 188.3, 162.7, 149.2, 139.0, 126.3, 122.8, 115.4, 63.9, 52.9, 50.5, 48.0, 29.6, 24.2, 19.8, 18.4.

### 6.3. Composites and Pellets

The composites were obtained by dissolving the suitable complex in ethanol and then adding the hydroxyapatite (1:1, *w*:*w*). The mixture was stirred for ca. 15 min and then the solvent evaporated under vacuum.

The pellets were obtained from the appropriate composite by shaping the optimized quantity (30 mg) of composite powder to form a thin pellet. All the pellets have the same diameter. A manual press was used to shape the pellets.

### 6.4. Cytotoxic Assays

#### Cell Lines, Cell Culture Conditions and Stock Solutions

The human osteosarcoma cell line MNNG/HOS (CRL-1547) was obtained from ATCC (American Type Culture Collection). For the experiments, cells were cultured in Dulbecco’s Modified Eagle’s Medium (DMEM with GlutaMAX™) (Thermo Fisher Scientific, Inc., Waltham, MA, USA), supplemented with 10% fetal bovine serum (FBS) and maintained at 37 °C in a 5% CO_2_ humidified atmosphere. The stocks of compounds’ solutions (10 mM) were prepared in DMSO, and serial dilutions from each stock were made in a complete medium (DMEM + FBS).

### 6.5. Cellular Viability

Cells were seeded in 96-well plates (2 × 10^4^ cells/200 µL medium) and incubated at 37 °C, 5% CO_2_ in a humidified atmosphere for 24 h to adhere. Then, the medium was discarded, and cells were incubated with the compounds at serial concentrations in complete medium in the range 0.1–100 µM for 24 h. After incubation, the cellular viability was determined using the colorimetric MTT assay as previously described [56]. This assay is based on the reduction of 3-(4,5-dimethylthiazol-2-yl)-2,5-diphenyltetrazolium bromide (MTT) by mitochondrial dehydrogenases in metabolically active cells to form purple formazan crystals. Briefly, after incubation with the complexes, the medium was discarded and 200 µL MTT solution in PBS (0.5 mg/mL) was added. After 3 h of incubation at 37 °C, the MTT solution was aspirated and 200 µL DMSO was added to solubilize the formazan crystals that formed inside the cells [57]. The IC_50_ was determined from dose–response curves using the GraphPad Prism 8.01 software.

The viability assays with the [Ag(I)]:HAP composites followed the same procedure as described above, incubating the cells with the composites at serial concentrations in complete medium in the range 0.1–50 µg Ag/mL for 24 h.

### 6.6. SEM Studies

Cells were seeded in 6-well plates (2 × 10^5^ cells/2 mL medium) over the HAP pellets and incubated at 37 °C, 5% CO_2_ in a humidified atmosphere for 24 h. After incubation, cells in the pellets were fixed by a four-step wash procedure as previously described [45]. Briefly, the medium was discarded, and cells were first washed with distilled water and fixed for three cycles with ethanol at different percentages to promote fixation. After drying, the pellets were analyzed by SEM using a Phenom ProX G6 apparatus (Thermo Fisher Scientific, Inc., Waltham, MA, USA).

### 6.7. Physicochemical Characterization

Powder X-ray Diffraction (PXRD)—data were acquired from powder samples of the complexes and composites using a D8 Advance Bruker AXS θ–2θ diffractometer (Bruker, Karlsruhe, Germany) with a LYNX EYE-XE detector, and a copper radiation source (Cu Kα, λ = 1.5406 Å), operated at 40 kV and 30 mA. Data were collected in the 5–50° 2θ range, with a step size of 0.05° and 0.5 s per step.

Scanning electron microscopy (SEM)—The images were acquired from powder of both complexes and composites and from pellets of composites using a Phenom ProX G6 apparatus (Thermo Fisher Scientific, Inc., Waltham, MA USA), and the elemental chemical composition was evaluated by the corresponding X-ray energy dispersive spectrometer (EDS).

### 6.8. Antibacterial Activity of the Complex and Composites

The antibacterial activity of complex **1** and the composites (**1A**–**4A** and **1B**–**4B**) towards *S. aureus* Newman, *E. coli* ATCC25922, *B. contaminans* IST408, and *P. aeruginosa* 477 was assessed by the determination of minimum inhibitory concentrations (MICs) using microdilution assays according to EUCAST (European Committee on Antimicrobial Susceptibility Testing) recommendations and as previously described [58]. *S. aureus* Newman (ATCC 13420) and *E. coli* ATCC25922 were obtained from the American Type Culture Collection. *Pseudomonas aeruginosa* and *Burkholderia contaminans* are from our own culture collection. Both trains were isolated from human infections [44]. In particular, *S. aureus* Newman was isolated from a case of secondarily infected tubercular osteomyelitis [58].

All the strains were maintained in Lennox Broth (LB) solid medium, composed of 10 g/L tryptone, 5 g/L yeast extract, 5 g/L NaCl, and 20 g/L agar. A minor modification of the protocol was performed for testing the composite antibacterial activity assay. Briefly, stock solutions of the composites were prepared in Mueller-Hinton broth (MHB, Sigma-Aldrich) at a final concentration of 80 µg Ag/mL. Serial 1:2 dilutions of stock solutions were prepared in 96-well microplates for each composite in MHB, the final concentrations being between 40 and 0.156 μg_Ag_/mL. Exponentially grown bacterial cultures were diluted with MHB fresh medium and added to the 96-well plates at a final inoculum of 5 × 10^5^ CFU/mL. The microplates were then incubated for 22 h at 37 °C. After incubation, the wells were examined for turbidity (growth), resuspended by pipetting, and their optical density was measured in a SPECTROstar Nano microplate reader (BMG Labtech) at 640 nm.

At least 3 independent experiments were performed in duplicate for each complex and composite under study. The MIC value was defined as the lowest concentration of the antimicrobial that inhibited the visible growth of a microorganism after the incubation time. Positive (no composites or complex) and negative controls (no inoculum) were performed for each experiment.

### 6.9. Antibacterial Activity of Pellets

The bacterial strains *Pseudomonas aeruginosa* 477 and *Staphylococcus aureus* Newman were used in this work and maintained in Lennox Broth (LB) solid medium. Both trains were isolated from human infections [44]. In particular, *S. aureus* Newman was isolated from a case of secondarily infected tubercular osteomyelitis [59].

To prepare the initial bacterial suspension, each strain was inoculated at a final optical density measured at λ = 640 nm (OD_640_) of 0.05 in 4 mL Mueller–Hinton broth (MHB, Sigma-Aldrich). After 5 h of incubation at 37 °C, the bacterial cultures were diluted to an OD_640_ of 0.025 in MHB. The sample pellets were gently added to 24-well cell culture plates with a flat bottom (CELLSTAR, Greiner bio-one) and 750 µL of the bacterial suspension was added. Wells without pellets samples were also tested to estimate the amount of bacterial colony-forming units (CFUs) without pellet samples. Wells containing only MHB (controls) were added to test the medium culture sterility.

After culturing the bacteria on 24 well plates for 42 h at 37 °C, the bacteria non-adherent and adherent to the pellet samples were quantified by serial dilution and spot-inoculation methods. For the non-adherent bacteria, the medium culture was serially diluted and plated onto the surface of LB agar plates for overnight culturing. For the adherent bacteria, the medium culture was removed, and the pellet samples were washed with 1 mL NaCl 0.9% (*w*/*v*). Then, the pellet samples were resuspended in 1 mL NaCl 0.9% (*w*/*v*), mechanically disrupted with a sterile tip, and vortexed for 1 min. The bacterial suspensions were serially diluted and plated onto LB agar plates for overnight culturing. The number of colonies on the plates was counted, and the number of colony-forming units of bacteria that were non-adhered and adhered to pellets was calculated.

For SEM analysis, the above protocol was performed for the pellet-adherent bacteria. However, the mechanical disruption of the pellet was not performed and the bacterial cells on the pellet were fixed as previously described [45].

### 6.10. Statistical Methods

All statistical analysis was performed using GraphPad Prism 8.0.1 software. Data are plotted as mean ± SD for all bar graphs. Figure legends indicate the test run, one-way ANOVA with indicated multiple comparison tests.

## Figures and Tables

**Table 1:** Cytotoxic activity ^a^ of Ag(I) camphorimine complexes based on ligands L^A^, L^B^ and L^C^ against HOS cells.

Compound	L	Y/R/Z	IC_50_ (µM) [IC_50_(μg_Ag_/mL)]
[Ag(NO_3_)(L^A^)_2_]	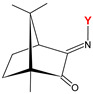	OH	8.2 ± 3.0 [0.88 ± 0.32]
[Ag(NO_3_)(L^A^)] **(1)**		NH_2_	5.6 ± 2.0 [0.60 ± 0.27]
[Ag(NO_3_)(L^A^)_2_]		C_6_H_5_	8.8 ± 2.5 [0.95 ± 0.22]
[Ag(NO_3_)(L^A^)_2_] **(2)**		*m*-OH(C_6_H_4_)	12.7 ± 4.5 [1.4 ± 0.48]
OC_10_H_14_NY ligand		NH_2_	>100
[Ag(NO_3_)(L^B^)_2_]	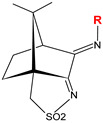	C_6_H_5_	8.3 ± 2.0 [0.89 ± 0.32]
[Ag(NO_3_)(L^B^)_2_]		C_6_H_4_NH_2_	23.7 ± 10 [2.6 ± 0.11]
[Ag(NO_3_)(L^C^)] **(3)**	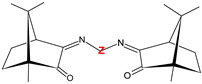	*p*-C_6_H_4_	3.1 ± 3.0 [0.30 ± 0.32]
[Ag(NO_3_)(L^C^)] **(4)**		*p*-(C_6_H_4_)_2_	3.1 ± 0.9 [0.33 ± 0.10]
AgNO_3_	—	—	7.2 ± 2.5 [0.78 ± 0.27]

^a^ Values upon 24 h exposure.

**Table 2:** Anticancer (IC_50_) and antibacterial (MIC) activity of Ag(I) camphorimine complexes (**1**–**4**) and [Ag(I)]:HAP composites ^a^.

	IC_50_	MIC			
	μg_Ag_/mL				
	HOS	*E. coli* ATCC25922	*B. contaminans* IST408	*P. aeruginosa* 477	*S. aureus Newman*
**1**	0.60 ± 0.27	2.3 ± 0.02	2.3 ± 0.1	1.6 ± 0.2	8.6 ± 0.02
**1A**	—	5.3 ± 0.6	6.6 ± 4.6	5.3 ± 0.2	40
**1B**	6.7 ± 3.8	4.7 ± 0.4	4.9 ± 0.6	4.2 ± 0.3	21.0 ± 0.8
**2** ^b,c^	1.37 ± 0.48	8.6	12.3	8.7	18.6
**2A**	—	5.8 ± 1.5	7.6 ± 0.7	5.5 ± 0.4	12.2 ± 1.5
**2B**	31 ± 4.5	20.1 ± 0.2	20.6 ± 2.5	21.7 ± 0.5	>40
**3** ^b^	0.33 ± 0.32	3.8 ± 0.2	6.8 ± 0.5	3.6 ± 0.8	13.7 ± 0.4
**3A**	—	8.4 ± 1.1	8.3 ± 5.3	4.1 ± 0.3	37.4 ± 3.6
**3B**	2.5 ± 0.7	5.2 ± 0.1	6.2 ± 0.4	3.8 ± 0.6	40
**4** ^b,c^	0.33 ± 0.10	>17	>17	14.3 ± 1.1	>17
**4A**	—	8.9 ± 0.1	9.0 ± 0.6	5.9 ± 0.1	40
**4B**	4.4 ± 1.2	4.7 ± 0.2	9.9 ± 1.3	3.5 ± 0.8	40
HAp	—	>630	>630	>630	>630
AgNO_3_ ^b^	0.78 ± 0.27	2.4 ± 0.1	4.0 ± 0.9	2.1 ± 0.4	19.1 ± 2.1

^a^ Composites [Ag(I)]:HAp (weight compositions): 20:80% (**1A**–**4A**) and 50:50% (**1B**–**4B**). ^b^ MIC values for complexes **2**–**4** and Ag(NO_3_) were taken from the literature [39,44]. ^c^ Values for **2** are shown as reported (no standard deviation).

## Data Availability

The raw/processed data required to reproduce these findings cannot be shared at this time due to technical limitations.
